# Derived from fangchinoline, LYY-35 exhibits an inhibiting effect on human NSCLC cancer A549 cells

**DOI:** 10.7150/jca.96582

**Published:** 2024-06-03

**Authors:** Bo Wang, Shan Long, Junjie Lan, Kaixiong Luo, Wangming Zhang, Xiaosong Li, Weidong Pan, Jielin Liu

**Affiliations:** 1 Department of Immunology, Basic Medical College, Guizhou Medical University, Guiyang, 550025, China.; 2 Oncology department, General Hospital of Hunan Medical College, Huaihua, 418000, China.; 3 Department of Pharmacy, Guizhou Provincial People's Hospital, Guiyang, 550002, China.; 4 School of Pharmaceutical Sciences, Guizhou University, Guiyang, 550025, China.; 5 Department of Oncology, The Seventh Medical Center of Chinese PLA General Hospital, Beijing, 100039, China.; 6 State Key Laboratory of Functions and Applications of Medicinal Plants, Guizhou Medical University, Guiyang, 550014, China.

**Keywords:** LYY-35, A549, cell cycle, cell apoptosis, DNA damage

## Abstract

Although fangchinoline has been widely used as an adjunct therapy for a variety of inflammatory and cancerous diseases, its mechanism of action on tumor cells remains unclear. Fangchinoline derivative LYY-35 reduced the number of A549 cells, deformed cell morphology and increased cell debris. Cell viability was significantly reduced, while the same concentration of LYY-35 had little effect on BEAS-2B viability of normal lung epithelial cells. In addition, LYY-35 can also reduce the migration, proliferation and invasion ability of A549 cells. Levels of β-catenin, ZO-1 and ZEB-1 proteins, biomarkers of cell adhesion and epithelial mesenchymal transformation, were significantly reduced. The levels of superoxide dismutase and lactate dehydrogenase decreased gradually, while the levels of glutathione, malondialdehyde and intracellular and extracellular ROS increased significantly. At the same time, LYY-35 induced increased apoptosis, increased expression of Bax, cleaved caspase3, cleaved PARP1, and decreased expression of Bcl-xl, which blocked the cell cycle to G0/G1 phase. The expressions of cell cycle checkpoint proteins Cyclin B1, Cyclin E1, CDK6, PCNA and PICH were significantly decreased. With the increase of LYY-35 concentration, the trailing phenomenon was more obvious in single cell gel electrophoresis. DNA damage repair proteins: BLM, BRCA-1 and PARP-1 expression decreased gradually.LYY-35 can inhibit the proliferation of non-small cell lung cancer A549 cells, block cell cycle, promote apoptosis, increase ROS production, cause DNA damage and interfere with DNA replication. LYY-35 is promising for the treatment of non-small cell lung cancer in the future.

## Introduction

The incidence of lung carcinoma has escalated into a predominant health issue, threatening individuals' health internationally 1. In the United States, lung cancer constitutes the primary cause of mortality attributed to cancer among both genders 2. The American Cancer Society forecasts that in 2023, there will be approximately 1,958,310 new cancer cases, of which 238,340 will be new instances of lung cancer, accompanied by an estimated 127,070 fatalities 3. Data from the tumor registry of the National Cancer Center of China indicate that lung cancer holds the highest incidence rate among malignant tumors in the country, based on the number of reported cases. In 2022, approximately 1,060,600 new lung cancer cases were recorded in China, corresponding to a crude incidence rate of 106.1 per 100,000 individuals. Based on mortality statistics, lung cancer was the leading cause of death from malignant tumors in China. In 2022, lung cancer was responsible for approximately 733,300 fatalities, corresponding to a mortality rate of 73.3 per 100,000 individuals 4. Lung cancer is categorically divided into two main types: small cell lung cancer (SCLC) and non-small cell lung cancer (NSCLC), with lung adenocarcinoma constituting the predominant subtype of NSCLC 5, 6. The contemporary therapeutic approaches for lung adenocarcinoma encompass surgical intervention, chemotherapy, radiotherapy, targeted therapy, and immunotherapy 7-9. Despite their efficacy, chemotherapy and radiotherapy are burdened by substantial side effects, and resistance to targeted therapy and immunotherapy frequently occurs 7, 9, 10. This underscores the imperative for the identification of new treatment modalities.

Fangchinoline B, a bisbenzylisoquinoline alkaloid isolated from the root of* Stephania tetrandra*, is a singular compound known for its diverse pharmacological properties 11. These include hypoglycemic, anti-proliferative effects on aortic smooth muscle cells, antihypertensive, and antioxidative activities 12. Investigations into fangchinoline have revealed its cytotoxic properties on several cancer cell types *in vitro*, encompassing cervical cancer Hela cells, breast cancer MCF cells, and cells from prostate, leukemia, gastric, and liver cancers. The compound is also known to inhibit proliferation and induce apoptosis within tumor cells 13-15. Nonetheless, the efficacy of this substance in suppressing human non-small cell lung cancer and its associated mechanisms warrant additional study.

Consequently, this study sought to explore the suppressive impact of LYY-35, a bioactive derivative of fangchinoline B, on A549 cells and to elucidate its underlying mechanism. The investigation revealed that LYY-35 exerted an inhibitory effect on A549 cells, with the extent of inhibition increasing concomitantly with the concentration of LYY-35. The mechanism underlying the action of LYY-35 appears to involve the suppression of cell proliferation, migration, and invasion, the enhancement of intracellular ROS, the induction of apoptosis, the inhibition of the cell cycle, and the disruption of DNA replication-related proteins. The data derived from this experiment are anticipated to provide essential research support for the application of LYY-35, thereby offering promising new treatment options for human non-small cell lung cancer patients.

## Materials and Methods

### Chemicals and reagents

**Figure 1:**
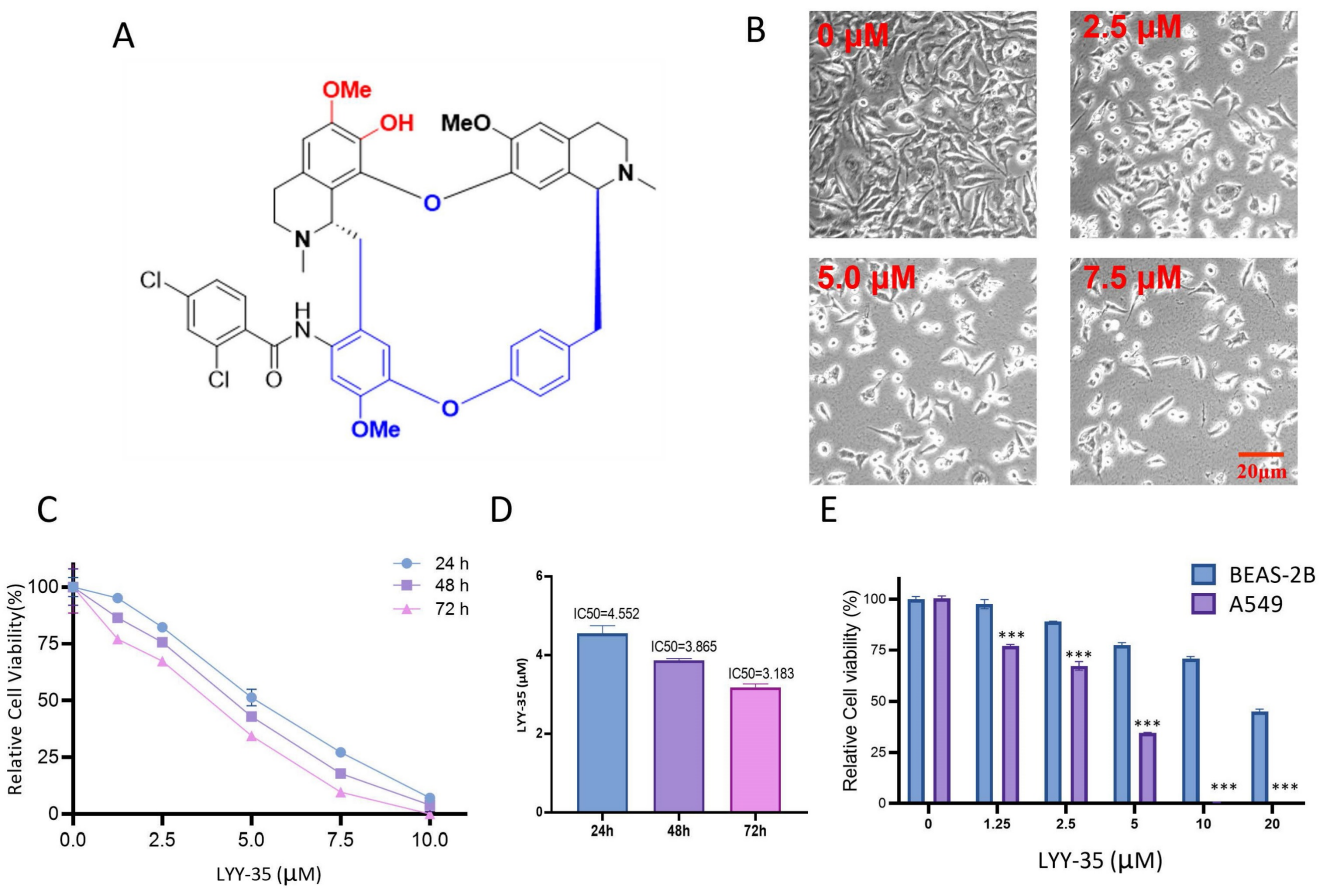
Effect of compound LYY-35 on cell viability. A. Chemical structure formula of LYY-35. B. A549 cells underwent treatment with LYY-35 for a duration of 24 hours, subsequent to which they were visualized and photographed using the microscope. C-D. An evaluation of cell viability metrics was conducted for A549 cells subjected to varying concentrations of LYY-35 over periods of 24, 48, and 72 hours. E. Effect of compound LYY-35 on the viability of BEAS-2B and A549 cells. BEAS-2B cells underwent treatment with varying concentrations of the compound LYY-35 (0, 1.25, 2.5, 5, 10, and 20 μM) for a duration of 72 hours, after which the IC_50_ value was determined utilizing GraphPad Prism software. Cell viability was quantified through the Cell Titer assay, with the reported result representing the mean of three replicates ± standard deviation (SD). *** *P* < 0.001.

LYY-35, a compound derived from *Stephania tetrandra*, was sourced from the Natural products research center of Guizhou province, characterized by a purity exceeding 95% and prepared in DMSO as the solvent. The molecular weight of LYY-35 is 795.24781, with its chemical structure depicted in Figure 1-A.

### Cell culture

Acquisition of the BEAS-2B normal bronchial epithelial cell line and the A549 human non-small cell lung cancer cell line was facilitated through the Chinese Academy of Sciences in Shanghai. The cell cultures of A549 and BEAS-2B were maintained in a controlled environment at 37°C and 5% CO_2_, utilizing penicillin-streptomycin (100 U/mL, HyClone)- and FBS (10%, Gibco)-contained DMEM.

### Assessment of cell viability

Following their progression to the exponential growth stage, A549 and BEAS-2B cells were distributed into 96-well plates, with each well containing 5,000 cells. Subsequent to an initial incubation of 12 hours, varying concentrations of LYY-35 (20, 10, 7.5, 5, 2.5, 1.25, and 0.625 µM) were introduced, followed by further incubation for durations of 24, 48, and 72 hours, respectively. The CellTiter-Glo® reagent was combined with the culture medium in a 1:1 ratio to prepare the working solution. Subsequently, 100 μL of this solution was dispensed into each well, ensuring thorough mixing. The assay plates were then shielded from light for a duration of 10 minutes, after which the luminescence intensity of each well was quantified utilizing a multifunctional microplate reader. The viability of cells in each group was quantified through comparison with the luminescence emitted by the blank control group. A dose-response curve correlating different concentrations of the drug with cell viability was constructed to facilitate the calculation of the drug's IC_50_ value.

### Hochest 33258 staining

In this experiment, 1×10^6^ A549 cells per well were distributed into 6-well plates and incubated with varying concentrations of LYY-35 (0, 2.5, 5.0, and 7.5 µM) for 48 hours. Following incubation, the cells were washed with PBS, fixed in 4% paraformaldehyde for 15 minutes, and subsequently stained with 1 μg/mL Hochest 33258 for a duration of 5 minutes. Subsequent to two PBS washes, the apoptotic features of the cancer cells were examined using confocal microscopy (SP8, Leica, Germany).

### Oxidative stress assay

Intracellular oxidative stress levels in response to varying concentrations of LYY-35 were quantified utilizing commercially available colorimetric assay kits. A549 cells, after treatment with diverse concentrations of LYY-35, were homogenized, and the supernatant was subsequently harvested for analysis. The concentrations of lactate dehydrogenase (LDH), glutathione (GSH), superoxide dismutase (SOD), and malondialdehyde (MDA) were determined using the A005 Glutathione Peroxidase Assay Kit, A001 Superoxide Dismutase Assay Kit, and A003 Microscale Malondialdehyde Assay Kit, with absorbance readings taken at wavelengths of 412 nm, 560 nm, and 532 nm, respectively. The quantification of extracellular ROS levels was conducted utilizing a ROS detection kit from Nanjing Jianchen. Conversely, intracellular ROS concentrations were determined through the staining of cells with 2,7-DCF-diacetate, employing a ROS assay kit provided by Beyotime Biotech, China. The resultant fluorescence was quantified by setting the excitation at 488 nm and measuring the emission at 525 nm.

### Wound healing motility assay and Transwell invasion assay

For the execution of the wound healing assay, A549 cell lines were distributed into 6-well plates, achieving a density of 5 × 10^5^ cells per well, and cultured to reach 90% confluence. An artificial wound was then introduced to the cell monolayer using a 200 μL pipette tip. The area of the wound was documented via an Olympus microscope at baseline and again 24 hours following the application of LYY-35.

The assessment of the invasive capabilities of A549 cells was conducted through the use of a transwell chamber, which was coated with Matrigel. In essence, the procedure entailed the addition of A549 cells to the chamber, utilizing a serum-free medium as the solvent for LYY-35. The lower chamber was then filled with complete medium, and incubation of the A549 cells ensued at 37℃ over a 48-hour. Cells that did not invade through the upper chamber were eliminated, and those that migrated beneath the membrane were subjected to staining with Wright's-Giemsa stain. Following staining, the cells were captured and quantified using a light microscope across a minimum of six randomly chosen fields.

### Western blot

Through the application of Western Blotting, the quantification of protein expression levels was achieved after lysing A549 cells subjected to treatments with LYY-35 at concentrations of 0, 2.5, 5.0, and 7.5 μM, leading to the collection and concentration determination of cellular proteins. Following separation on 4-12% sodium dodecyl sulfate-polyacrylamide gels, the total protein from the samples was transferred onto polyvinylidene difluoride membranes (Merck Millipore, Billerica, MA, USA) using a wet electrophoresis technique. Before the membranes were left to incubate with an array of primary antibodies (GAPDH, ZO-1, ZEB-1, β-catenin, cleaved caspase-3, Bax, Bcl-xl, CDK6, Cyclin B1, Cyclin E1, PICH, PCNA, cleaved PARP-1, PARP-1, BRCA-1, and BLM) at 4˚C throughout the night, 1-hour blocking using 5% skimmed milk was carried out. Post-wash with TBS, incubation was carried out for one hour at room temperature, utilizing goat anti-rabbit IgG antibodies that were conjugated to horseradish peroxidase. In the final stage of the procedure, detection of the protein bands was facilitated using an enhanced chemiluminescence system (Applygen Technologies Inc., Beijing, China), followed by the quantification of their gray values via Image J analysis software (National Institutes of Health, Bethesda, MD).

### Flow cytometry

Apoptosis in A549 cells, treated with varying doses of LYY-35, was quantified using flow cytometry. This quantification utilized the Annexin V-FITC/PI (propidium iodide) apoptosis detection kit from BD Biosciences Clontech. For the purpose of cell cycle analysis, treated A549 cells underwent fixation in 70% ethanol for an overnight period at -20°C, followed by processes of resuspension and digestion with RNase A. Propidium iodide (PI) at a concentration of 50 μg/mL was used to stain the DNA under dark conditions at 37°C for a period of 30 minutes. The process was followed by analytical evaluation of the stained cells with FACScan flow cytometry, utilizing CellQuest analysis software (Becton Dickinson, San Jose, CA).

### TUNEL assay

Employing terminal deoxynucleotidyl transferase mediated nick end labeling, the TUNEL assay was executed by following the protocol specified in the in-situ cell death detection kit's manufacturer instructions. Briefly, after harvesting of the LYY-35-exposed A549 cells, fixation using 4% formaldehyde and permeabilization the cells on the slide glasses were carried out. To detect apoptosis, the 3'-OH ends of fragmented DNA were labeled with biotin-dNTP, using the Klenow fragment at 37°C for a duration of 1.5 hours. For visualization, cell nuclei were counterstained using 4',6-diamidino-2-phenylindole (DAPI), with the resulting images acquired through the application of fluorescence microscopy.

### EdU labeling

For the purpose of assessing DNA synthesis and, consequently, cell proliferation, 5-ethynyl-2'-deoxyuridine (EdU) was employed as a labeling agent. EdU, acting as a thymidine analog, allows for incorporation into the DNA of cells in the process of division. The BeyoClick™ EdU Cell Proliferation Kit, integrated with Alexa Fluor 488 (Beyotime, Nanjing, Jiangsu, China), was used to perform EdU staining. Subsequently, Hoechst 33342 solution was utilized to counterstain the cell nuclei for a duration of 10 minutes at ambient temperature. Following this, the proportion of EdU-positive cells was quantified and subjected to comparative analysis.

### Comet assay

The comet assay, as described by Hartmann *et al.*, was employed for the analysis of cellular DNA breakage at the single-cell level. Briefly, A549 cells, following treatment with varying concentrations of LYY-35, were subjected to trypsinization, centrifuged at 250 g for 3 minutes, and subsequently resuspended. Subsequently, the A549 cells were treated with low-melting-point agarose and applied to microscopic slides that had been pre-coated with normal-melting-point agarose, followed by covering with a cover slip. These slides were then subjected to incubation in lysis buffer and underwent electrophoresis under conditions of 300 mA and 25 V for a duration of 30 minutes. Post-electrophoresis, the cell nuclei were counterstained with PI, and comet assay images were quantitatively evaluated using a wavelength range of 515-560 nm.

### Statistical analysis

Statistical examination of the data was carried out utilizing GraphPad Prism software, version 8.0, in conjunction with SPSS software, version 24.0. Data conforming to a normal distribution, as confirmed by the Shapiro-Wilk test, were expressed as the mean ± standard error of the mean, derived from three independent experiments conducted in triplicate. The statistical analysis was undertaken using either Student's t-test or one-way analysis of variance (ANOVA), contingent upon the data's requirements. Calculation of the inhibitory concentrations, specifically IC_25_, IC_50_, and IC_75_, was performed using non-linear regression analysis facilitated by the ED50plus software, version 1.0. The criterion for establishing statistical significance was a two-sided P-value of less than 0.05.

## Results

### LYY-35 treatment compromises A549 cells viability

The examination of A549 cells treated with LYY-35, regarding their morphological features and cell count, was performed via bright field microscopy. This examination confirmed that cell morphology was adversely affected, with an increase in cell fragmentation and mortality, alongside a decrease in the population of live cells (Figure 1-B). The cell tilter assay demonstrated a decrease in cell viability proportional to the increase in drug concentration (Figure 1-C). Furthermore, the IC_50_ value exhibited a reduction with an extended duration of drug exposure (Figure 1-D). Notably, the cytotoxic effects of LYY-35 on BASE-2B cells were significantly lower than those observed in A549 cells at equivalent concentrations (Figure 1-E). These findings suggest that LYY-35 markedly reduces tumor cell viability, exhibiting greater cytotoxicity compared to normal cells.

### Effects of LYY-35 on cellular oxidative stress

**Figure 2:**
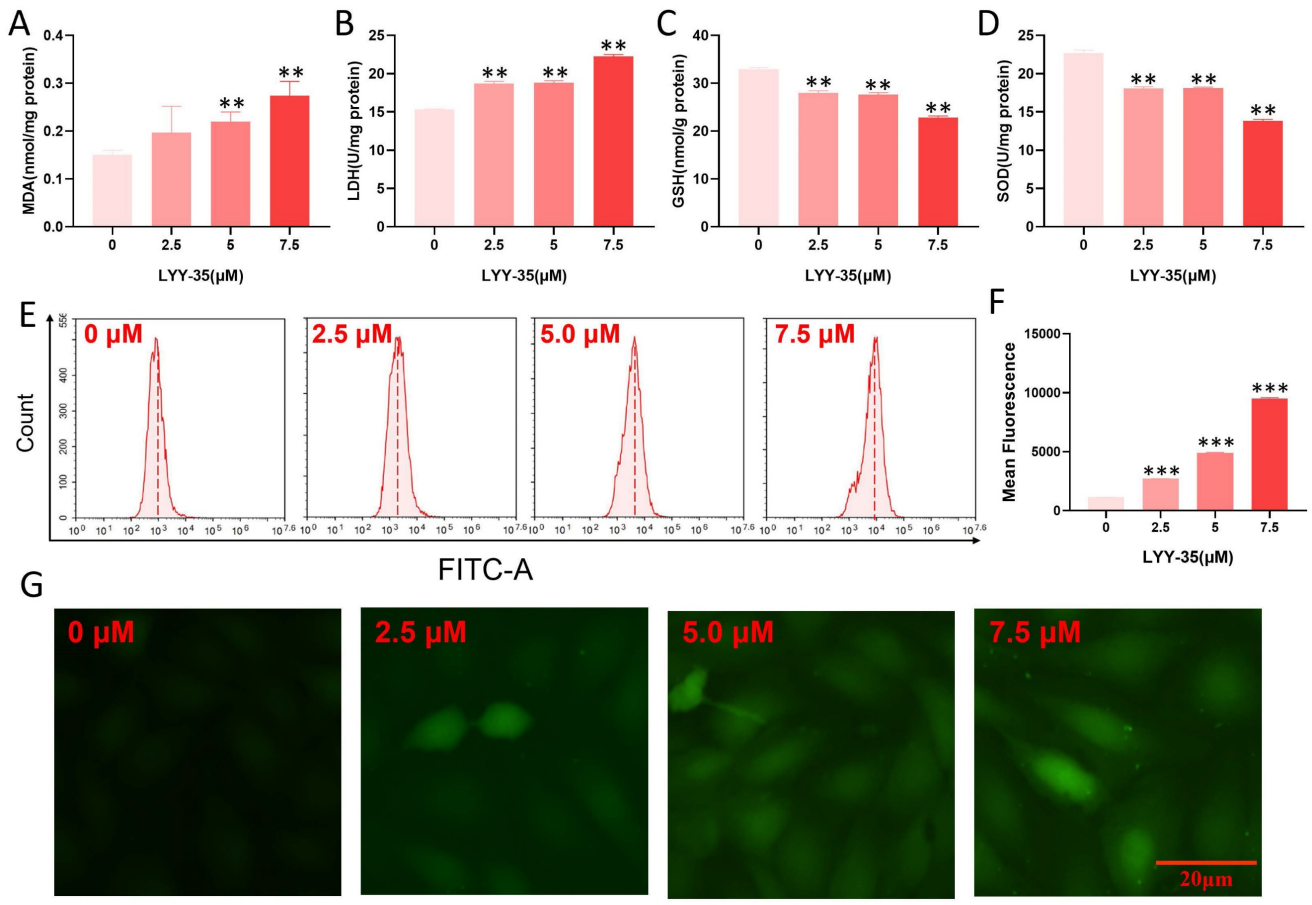
Oxidative damage of A549 cells by LYY-35. A-D. Illustrate the expression levels of MDA, SOD, LDH, and GSH in A549 cells following treatment with LYY-35 at incremental concentrations of 0, 2.5, 5.0, and 7.5 μM, respectively. E. Illustrations of ROS activity subsequent to administration of LYY-35 in dosages of 0, 2.5, 5.0, and 7.5 μM. F. ROS statistical column analysis chart. Quantification of intracellular ROS following LYY-35 administration. G. Changes of intracellular ROS fluorescence intensity. The outcome is an average of three replicates, with variability expressed as standard deviation (SD). ** *P* < 0.01, *** *P* < 0.001.

The impact of LYY-35 on ROS in A549 cells was investigated utilizing flow cytometry and a cell oxygen activity probe assay. Upon increasing exposure to LYY-35, a significant augmentation in the levels of MDA and GSH was observed, accompanied by a pronounced decline in the levels of SOD and LDH (Figure 2A-D). Furthermore, analyses conducted via flow cytometry and fluorescence imaging using the DCF probe revealed that intracellular levels of ROS escalated in response to increasing concentrations of LYY-35 (Figure 2E-G). Collectively, these findings demonstrate that treatment with LYY-35 enhances ROS production in A549 cells, with the extent of ROS-induced damage intensifying alongside higher drug concentrations.

### Effects of LYY-35 on cell migration, invasion and proliferation

**Figure 3:**
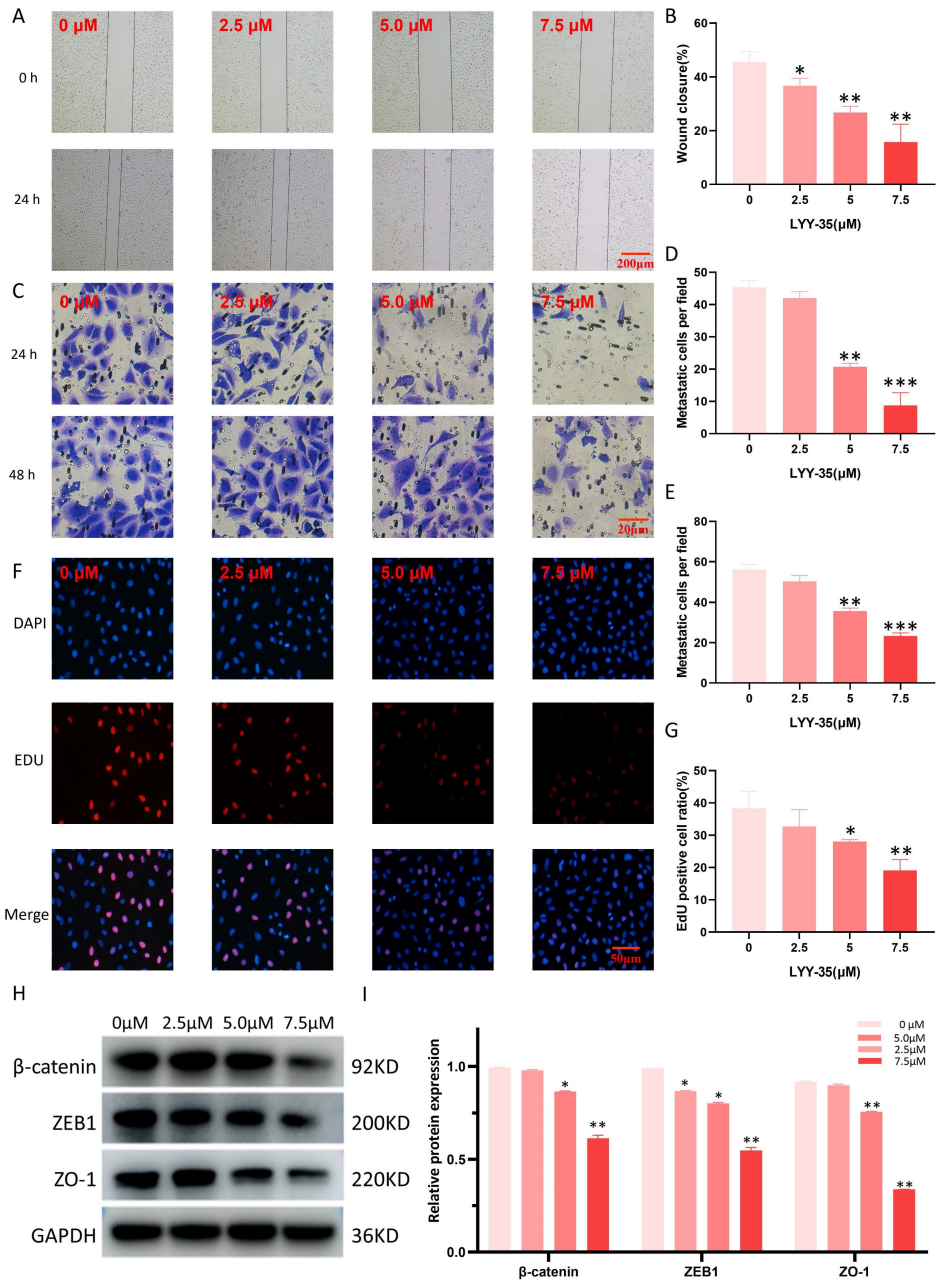
Effects of LYY-35 on proliferation, invasion and migration of A549 cells. A-B. Scratch experiment. C-E. Transwell experiment. F-G. Fluorescence staining. H-I. Results of the Western Blot (WB) experiment and the grayscale value columnar analysis. Data represent the mean of three replicates, expressed as ± standard deviation (SD). * *P* < 0.05, ** *P* < 0.01, *** *P* < 0.001.

The assessment of LYY-35's impact on A549 cell migration and invasion was performed through the application of scratch and Transwell assays. LYY-35 was found to diminish the ability of A549 cells to migrate and invade in a manner proportional to the dosage (Figure 3A-E). The capacity for cell proliferation can be assessed through EDU experiments. Treatment with LYY-35, administered in a gradient concentration, inhibited the proliferation of A549 cells. Specifically, a marked decrease in proliferation was evident at the concentration of 7.5 μM (Figure 3F-G). Simultaneously, the expression levels of proteins associated with EMT in the cells underwent significant changes. Treatment of A549 cells with LYY-35 for 24 hours resulted in a marked decrease in the concentrations of β-catenin, ZO-1, and ZEB-1, key biomarkers of cell adhesion and epithelial to mesenchymal transition (Figures 3H-I). Evidence from these results shows that LYY-35 substantially impedes cellular processes involving migration, proliferation, and invasion.

### Effects of LYY-35 on cell cycle

**Figure 4:**
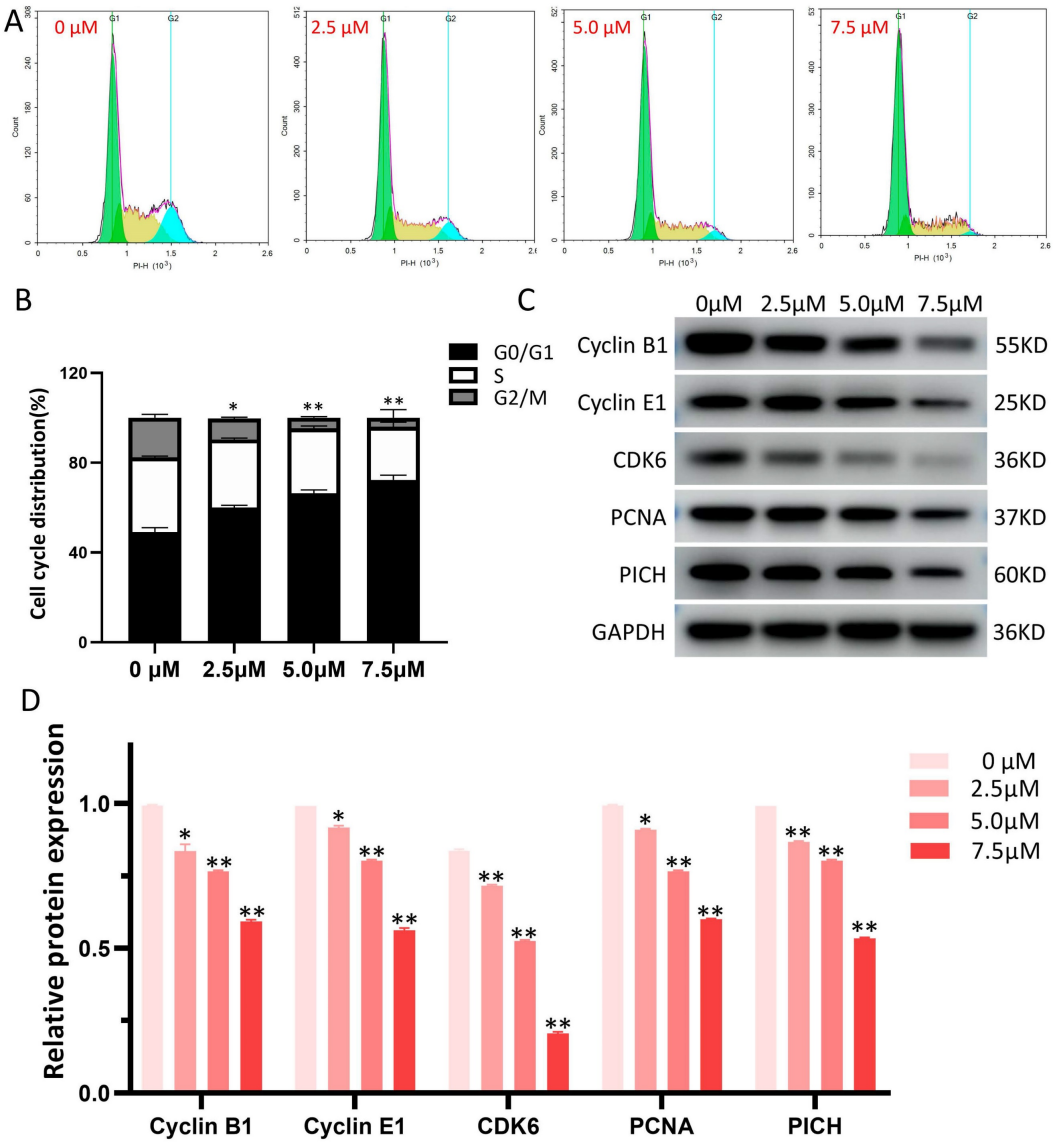
LYY-35 triggers cell cycle arrest in A549 cells. A. The application of flow cytometry indicated that treatment with LYY-35 at concentrations of 0, 2.5, 5.0, and 7.5 μM resulted in cells being allocated to distinct stages within the cell cycle. B. The grayscale values obtained from flow cytometry were quantified utilizing GraphPad Prism software. C. The impact of LYY-35 on the expression of Cyclin B1, Cyclin E1, CDK6, PCNA and PICH proteins in A549 cells was demonstrated through Western blotting. The analysis was conducted on three separate occasions to confirm the results. D. The grayscale values of the Western blot were determined utilizing GraphPad Prism software, with the outcomes expressed as a mean ± SD from three experimental replicates. * *P* < 0.05, ** *P* < 0.01.

The influence of LYY-35 on the cell cycle of A549 cells was quantified using flow cytometry, revealing that LYY-35 elicited a dose-dependent inhibition of cell cycle progression (Figure 4A). The arrest of the cell cycle was documented at the G0/G1 stage (Figure 4B), accompanied by notable changes of cell cycle checkpoint proteins' expression. With increasing concentrations of LYY-35 over a 24-hour treatment period, there was a significant reduction in the concentrations of Cyclin B1, Cyclin E1, CDK6, PCNA, PICH, among other cyclin proteins in A549 cells (Figure 4 C-D). This demonstrates that LYY-35 significantly impedes the cell cycle in A549 cells.

### Effect of LYY-35 on cell apoptosis

**Figure 5:**
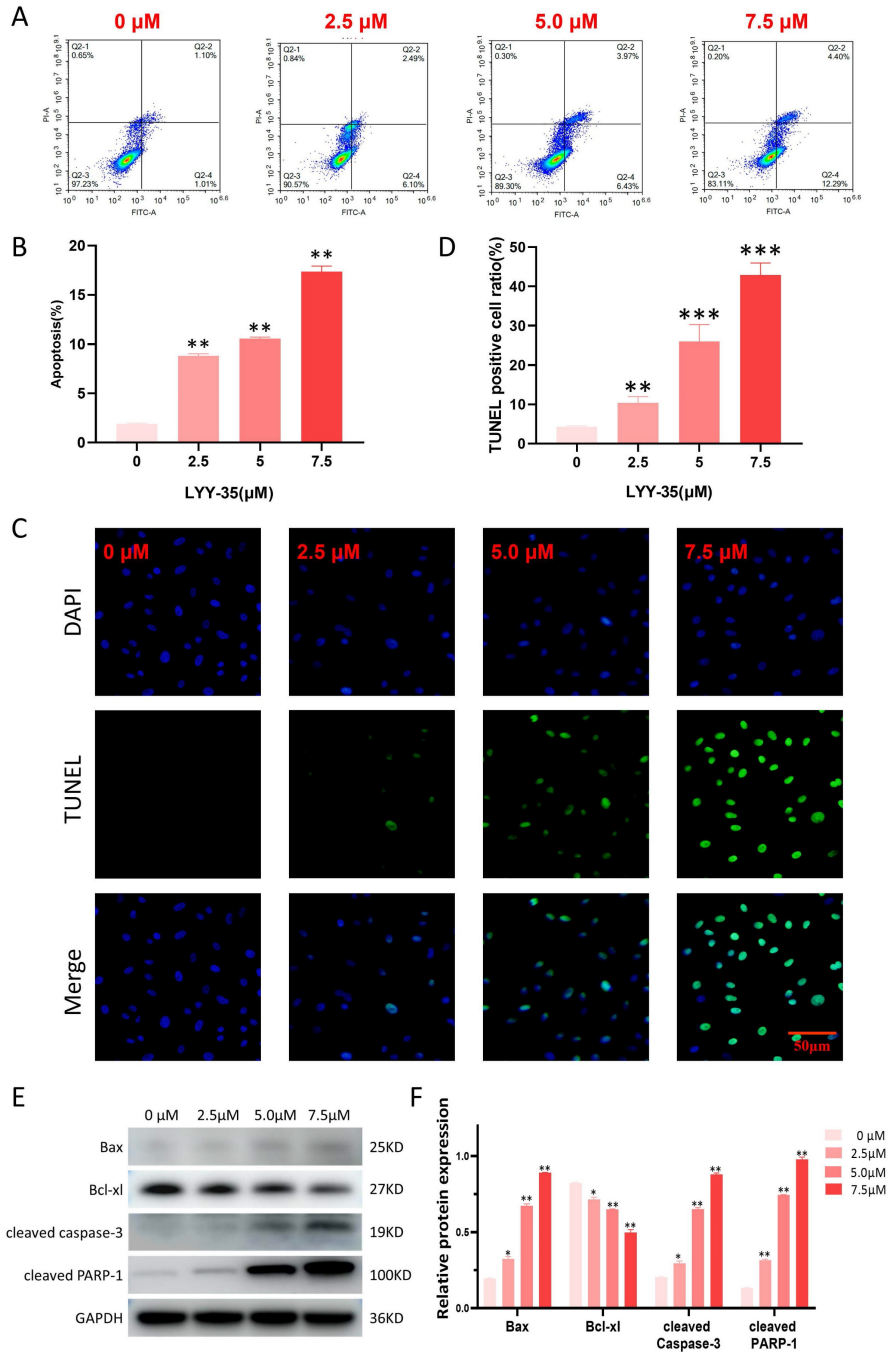
Effect of LYY-35 on apoptosis of A549 cells. A-B. Quantification of apoptosis and the generation of apoptosis histograms through flow cytometric analysis. C-D. The positive ratio determined by the TUNEL assay demonstrated an increase concomitant with escalating concentrations of the drug. E-F. Assessment of apoptotic protein expression and grayscale quantification histogram. Data represent the mean of three replicates ± standard deviation (SD). * *P* < 0.05, ** *P* < 0.01, *** *P* < 0.001.

In the current investigation, flow cytometry was employed to assess the impact of LYY-35 on apoptosis within A549 cells. The findings revealed a concentration-dependent increase in the proportion of apoptotic cells (Figure 5A-B). The TUNEL assay, which is capable of identifying DNA fragmentation—a hallmark of apoptotic cells—demonstrated a significant elevation in the proportion of TUNEL-positive cells following treatment with LYY-35 (Figure 5C-D). An increase in LYY-35 concentration was associated with the upregulation of proteins related to cell apoptosis, including the pro-apoptotic protein Bax, cleaved PARP-1, and cleaved caspase 3. There was a notable reduction in the expression of the anti-apoptotic protein Bcl-xl (Figures 5E-F). Consequently, it can be concluded that LYY-35 effectively induces apoptosis within A549 cells.

### Effects of LYY-35 on DNA damage

**Figure 6:**
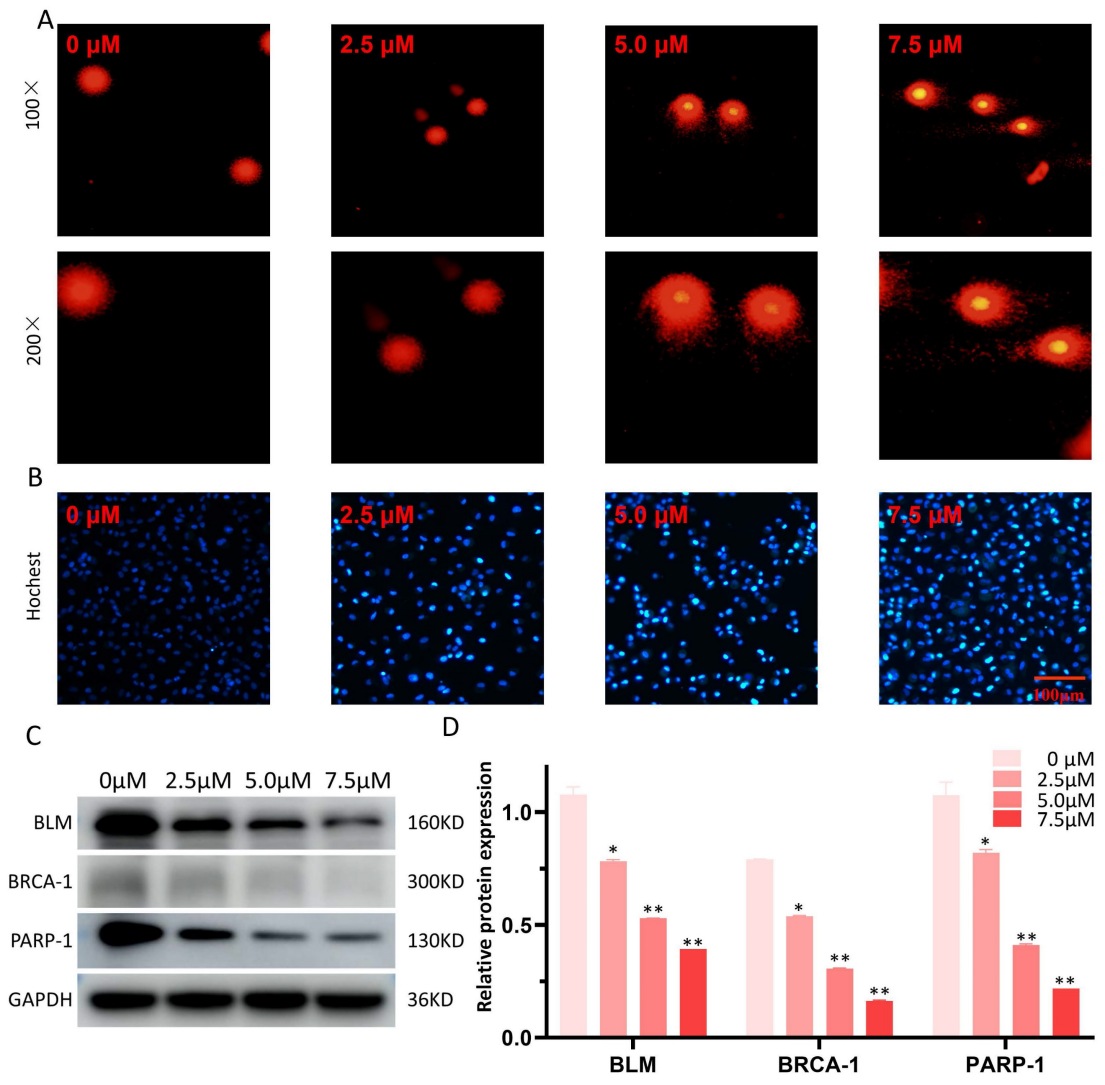
Effect of LYY-35 on DNA damage in A549 cells. A. The comet alkaline assay, a form of single cell gel electrophoresis, demonstrates that higher concentrations are associated with more severe DNA damage in cells, manifesting as more distinct comet tailing. B. Hochest test was performed after the cells were treated with gradient concentration of LYY-35. C-D. The trend of the target protein as observed in the Western blot (WB) experiment and the columnar analysis diagram. Results represent the mean of three replicates ± standard deviation (SD). * *P* < 0.05, ** *P* < 0.01, *** *P* < 0.001.

The Comet alkaline assay (single cell gel electrophoresis) was employed to evaluate DNA damage attributable to LYY-35. Observations indicated an enhancement in DNA damage, manifesting as more pronounced comet tails, concomitant with increases in LYY-35 concentration (Figure 6A). Additionally, Hochest staining corroborated the concentration-dependent augmentation of DNA damage (Figure 6B). Furthermore, a decrease in the expression levels of DNA repair proteins, including BLM, BRCA-1, and PARP-1, was observed (Figure 6C), suggesting that LYY-35 induces DNA damage in A549 cells.

## Discussion

Derived from *Stephania tetrandra*, fangchinoline is a natural compound characterized by its small molecular structure and diverse biological activities. Historically, it has served as an adjunctive therapy in the management of various inflammatory and autoimmune diseases 11, 12, 15. Previous research indicates that fangchinoline exhibits cytotoxic effects on Hela cervical cancer cells, MCF breast cancer cells, and has demonstrated efficacy in *in-vitro* studies of prostate cancer, leukemia, gastric cancer, and liver cancer. This compound is reported to inhibit tumor cell proliferation and facilitate apoptosis 12-15. However, a comprehensive understanding of the precise molecular mechanisms involved remains largely elusive. This study established that LYY-35 possesses cytotoxic properties against A549 cells, which intensify with escalating concentrations of the drug. Additionally, comparative analysis indicated that normal lung epithelial cells experienced lower levels of cytotoxicity from the same concentrations of LYY-35 than A549 cells. Earlier studies have documented that the indiscriminate cytotoxicity exhibited by chemotherapeutic agents towards normal lung tissue may contribute to a range of side effects, such as acute lung injury and bone marrow suppression (myelosuppression) 16-19. Thus, there is a potential for LYY-35 to serve as an effective therapeutic option for lung cancer.

This study further substantiated that LYY-35 exhibits the capacity to impede the migration, proliferation, and invasion of A549 cells. Specifically, a strong correlation has been identified between the migratory and invasive capabilities of cancer cells and the development of metastases, which frequently serve as predictors of treatment failure and adverse prognoses 20. The influence of LYY-35 on the migration, proliferation, and aggressiveness of A549 cells underscores its potential utility as a biomarker for forthcoming clinical interventions. ZO-1, a protein integral to the formation of tight junctions and necessary for cell adhesion, has been identified by previous studies as playing a fundamental role in the metastatic dissemination of tumors, with reduced expression marking a significant step in this process 21. ZEB-1 protein significantly contributes to the modulation of the EMT process. Decreased levels of ZEB-1 protein are associated with the inhibition of migration and proliferation in tumor cells, leading to reduced tumor growth 22. The PICH protein, functioning as a DNA translocation enzyme, is critical for the disassembly of chromosome bridges during mitosis. A reduction in PICH levels is correlated with heightened chromosomal instability, leading to increased cellular mortality 23. PCNA expression is directly correlated with the proliferation of cells. Specifically, in neoplastic cells, a heightened expression of PCNA has been observed to significantly enhance the rapid proliferation of tumor masses 24, 25. Beta-catenin, functioning as a scaffold molecule, predominantly resides within cadherin-based adhesion junctions. A diminution in the expression of beta-catenin has been demonstrated to facilitate metastasis in patients with lung cancer, correlating with an adverse prognostic outlook 26. Consequently, within the scope of this study, the observed reduction in the protein levels of ZO-1, ZEB-1, PICH, PCNA, and β-catenin following stimulation with LYY-35 substantiates the hypothesis that LYY-35 possesses the capability to attenuate invasion, proliferation, and metastasis in A549 cells.

Currently, apoptosis serves as a crucial metric for assessing the impact of LYY-35 on A549 cells. Techniques such as Western blotting, TUNEL assays, and flow cytometry have collectively demonstrated that the rate of apoptosis escalates in correlation with increasing concentrations of LYY-35 exposure. Apoptosis is contingent upon the activity of proteins within the caspase family, notably caspase 3, which undergoes activation within apoptotic cells and facilitates the targeted cleavage of numerous pivotal proteins associated with apoptosis, including cleaved PARP-1 27, 28. Bax and Bcl-xl are both constituents of the BCL-2 protein family, yet they exhibit antagonistic roles in the regulation of apoptosis. Specifically, the Bax protein interacts with several anti-apoptotic proteins, such as Bcl-xl. Upon activation, Bax relocates to the mitochondrial outer membrane, enhances its permeability, and consequently initiates apoptosis 29. Western blot analysis revealed that an increase in LYY-35 concentration correlates with an upregulation in the expression of cleaved caspase-3, cleaved PARP-1, and Bax protein, while concurrently observing a decrease in the expression of Bcl-xl protein. These findings collectively suggest that LYY-35 may enhance the apoptosis of tumor cells.

Additionally, LYY-35 exerts effects on the cell cycle, the arrest of the tumor cell cycle constitutes a critical factor in LYY-35's ability to impede tumor proliferation.

Flow cytometric analysis post-propidium iodide (PI) staining revealed that tumor cells experienced stagnation at the G0/G1 phase. The sustained proliferation of tumor cells is contingent upon uninterrupted progression through the cell cycle 30. Consequently, each stage of the cell cycle plays an essential role in maintaining homeostatic balance. Interruption of specific cell cycle phases can lead to a decrease in cell division rates and subsequently impede tumor proliferation 30. Upon administering LYY-35 to A549 cells, an investigation into the mechanism through which LYY-35 inhibits cell proliferation was conducted. Results indicated a dose-dependent increase in the size of cells in the G1/0 phase, along with a reduction in the proportion of G2/M phase cells from 17.94% to 1.37%. These findings imply that LYY-35 is capable of arresting the G1/0 cell cycle in A549 cells. Two critical components of cell cycle regulatory mechanisms are cyclins and cyclin-dependent protein kinases 31. CDK6 is known to interact with Cyclin E1 and Cyclin D, forming complexes that facilitate the transition of cells from the G1 phase to the S phase 32. Specifically, Cyclin E1, which is synthesized during the G1 phase, partners with CDK6 to constitute a complex that catalyzes the cell's progression into the S phase. Conversely, Cyclin B1, present during the S and G2 phases, associates with CDK1 to create a complex that orchestrates the cell's advancement from the G2 phase to the M phase 33. A decrease in the expression of CDK6, Cyclin B1, and Cyclin E1 leads to cell cycle arrest in the G0/G1 phase, impeding entry into the S phase and subsequently disrupting the completion of the full cell cycle, thereby affecting cellular processes vital for cell survival.

DNA damage represents another plausible mechanism underpinning the efficacy of LYY-35 against A549 cells. Numerous chemotherapeutic agents utilized in lung cancer treatment, including first-line therapies such as alkylating agents, platinum-based compounds, and topoisomerase inhibitors, exert clinical effectiveness by disrupting DNA replication in tumor cells. This interference not only inhibits the rapid proliferation of tumor cells but also compromises the integrity of the tumor genome, culminating in DNA damage 34, 35. The BLM and BRCA-1 proteins play pivotal roles in preserving DNA integrity and stability. The BLM protein is instrumental in unwinding the double-helical structure of DNA, mitigating excessive sister chromatid exchange, and facilitating the requisite spatial conditions for DNA repair and replication. Concurrently, the BLM protein participates in various DNA repair pathways, such as homologous recombination and non-homologous end joining, thereby ensuring genomic stability 36, 37. The BRCA-1 protein, functioning as a pivotal tumor suppressor, is integral to various cellular processes, including DNA repair, cell cycle regulation, and homologous recombination, among others 36. PARP-1, an essential enzyme in the repair of nucleic acids, identifies DNA single-strand breaks and orchestrates the assembly of repair proteins, thereby initiating the repair process through the catalysis of poly (ADP-ribose) synthesis 36-38. Treatment of A549 cells with LYY-35 resulted in a concentration-dependent decrease in the expressions of BLM, BRCA-1, and PARP-1 proteins, suggesting that LYY-35 may disrupt DNA replication in tumor cells, leading to DNA damage and compromising DNA stability. Consequently, this disruption impedes correct cell division, culminating in cell death. Furthermore, comet alkaline assay results corroborated this hypothesis, showing an intensified tails in DNA electrophoresis with increasing concentrations of LYY-35, indicative of DNA damage. The downregulation of critical DNA repair proteins, including BLM, BRCA-1, and PARP-1, hinders effective DNA repair, thereby affecting cell proliferation and apoptosis.

## Conclusion

The fangchinoline derivative, LYY-35, significantly decreased the viability of A549 cells while exhibiting reduced toxicity towards normal cells. It effectively inhibited cell migration, proliferation, and invasion, stimulated the generation of ROS, and suppressed tumor cell growth. DNA damage and apoptosis are postulated as the underlying mechanisms contributing to its cytotoxic effects. These findings offer new insights into the molecular actions of LYY-35 and highlight its potential utility as a chemopreventive agent in lung cancer treatment. Nevertheless, to conclusively determine its efficacy and elucidate its mechanisms of action, further studies involving *in vivo* models in animals and clinical trials in humans are warranted.
